# Friendship Quality and Gender Differences in Association With Cyberbullying Involvement and Psychological Well-Being

**DOI:** 10.3389/fpsyg.2019.01723

**Published:** 2019-07-24

**Authors:** Mairéad Foody, Lian McGuire, Seffetullah Kuldas, James O’Higgins Norman

**Affiliations:** National Anti-Bullying Research and Resource Centre, Dublin City University, Dublin, Ireland

**Keywords:** cyberbullying, friendship quality, gender, psychological well-being, post-primary

## Abstract

Current literature has documented the detrimental effects of cyberbullying which include a range of internalizing and externalizing problems for those involved. Although critical, this research can sometimes ignore social-ecological aspects of a child’s life that can potentially ‘buffer’ the negative psychological effects of such involvement. With this in mind, this cross-sectional investigation of 12–16 year olds [M(SD): 13.5(1) years] in Ireland focused on the role of friendship quality and gender in association with cyberbullying involvement and psychological well-being (*N* = 2410). The Cyberbullying and Online Aggression Scale was used to measure cyber perpetration and victimization. A modified version of the Cambridge Friendship Questionnaire was included to investigate peer friendship quality. Finally, the Moods and Feeling Questionnaire and the Strengths and Difficulties Questionnaire were chosen to provide a measurement of psychological well-being. Prevalence rates for various types of cyberbullying roles (cyber bullies, victims and bully/victims) are presented, as well as differences for psychological well-being, friendship quality and cyberbullying involvement. In addition, regression models were used to determine the associations between gender, age, friendship quality and involvement in cyberbullying with psychological well-being. The results are considered in terms of the current literature and directions for future research are suggested.

## Introduction

Whilst the advantages of digital technology are of great use to adolescents and have been widely embraced, the increasingly ubiquitous use of online technologies has also brought with it increased risk in the form of cyberbullying (Cross et al., 2009). Cyberbullying has been defined as ‘negative or hurtful, repetitive behavior, by the means of electronic communication tools, which involve an imbalance of power with the less-powerful person or group being unfairly attacked’ (Smith et al., 2008). Common forms involve relational and verbal bullying, including the distributing of rumors and/or hurtful comments, the issuing of images, threats, or disclosure of true or false personal information via phone text messages email, websites, gaming or social networking sites (e.g., Twitter, Facebook, YouTube, Snapchat).

Contextual factors in regards to cyberbullying, as with most stressors, are key, and studies have found that different forms of cyberbullying may elicit differing emotional responses. For example, being bullied online may evoke a different emotional response than being bullied via text (Ortega et al., 2009). Furthermore, there are types of cyberbullying that are perceived as less harmful than traditional bullying, such as insults and threats, while other forms (e.g., where pictures or videos are used/shared or where there is a perception of high risk of personal injury such as blackmail) may be considered more damaging (Smith et al., 2008). Of note, is that traditional and cyber acts of bullying can also be intertwined, with face to face conflict leading to issues online, or vice versa (Kowalski et al., 2008; Gleeson, 2014).

Unlike traditional forms of bullying, cyberbullying of teenagers relies on the direct provision and use of tools, i.e., hardware and internet access to teens most commonly provided by parents. These tools help to provide anonymity, making it harder to control; harder to remove due to proliferation on networks that redistribute the content; and allowing the cyberbullying to invade the adolescent’s personal space (e.g., home, downtime) in a way that traditional bullying cannot (O’Moore, 2014). In addition, cyberbullying may not directly involve the school environment, while trying to deal with social networks, phone companies and authorities can be complex and intimidating. As a result, it is to be expected that a greater onus for coping and support may fall more frequently upon the teenager’s own coping mechanisms and their personal social network (e.g., friends).

### Gender

Prevalence rates of cyberbullying amongst teens vary widely globally, ranging from 10 (Smith et al., 2008), to 20% (Garett et al., 2016) to up beyond 70% (Selkie et al., 2016) and there are mixed findings when it comes to gender involvement in cyberbullying. Some studies have reported that across a range of educational settings females are more likely than males to be involved in cyberbullying as a victim (Marcum et al., 2012; Beckman et al., 2013; Heiman and Olenik-Shemesh, 2015; Smith et al., 2019) In contrast, the amount of females versus males involved in cyberbullying perpetration shows great variation across studies with some reporting no significant differences (e.g., Mishna et al., 2010). There are many reasons why this may be the case, one of which was noted recently by Smith (2019) as being age, where the early adolescence period showed more females than males as perpetrators and the opposite to be the case for later adolescence. In addition, there is some evidence to suggest that there are gender differences in the way young people use the internet and ultimately the methods used to cyber bully. For example, girls are more likely to use the internet to talk to friends and share pictures, while boys are more likely to play video games (e.g., Mishna et al., 2010). Also, given that gender is often understood as a socially constructed term, this may influence how research participants respond to questions about gender. For the purposes of the current study we understand the term gender to relate to the sex of the participants (i.e., male or female).

Smith et al. (2019) highlighted the importance of factoring in cultural and sociological contexts when considering gender and cyberbullying. One recent study found that males are more involved in cyberbullying perpetration with the greatest gender difference in Asian countries, followed by North America, and it was least in European countries and Australia (Sun et al., 2016). In order to elaborate on why gender differences may occur in bullying behavior we can also look to both dispositional and structural explanations of different social behaviors observed among males and females offline and online. Dispositional approaches provide an understanding of male and female behavior arising from biological and early-life cultural socialization, whereas structural approaches explain the differences by the positions male and females take in society and their differential access to political, economic and ideological resources (Fischer and Olicker, 1983). Some significant differences have been observed between friendships among girls and those among boys, arising from socially constructed gender norms. Girls’ friendships have been observed to be more intensive and intimate than those of boys, and usually involve a limited number of girlfriends whereas boys are socialized to be autonomous and goal-oriented. Girls’ socialization and positive sense of self is very much focussed on relationships and empathetic connectedness. In this sense, threats to relationships can also be experienced as a threat to girls’ sense of self. This suggests that (gender-normative) girls have a greater vested interest than (gender-normative) boys in maintaining friendships and resolving conflict (Ging and O’Higgins Norman, 2016). In this context, we are interested in exploring the extent to which gender influences friendship quality and how girls and boys manage experiences of cyberbullying.

### Effects on Well-Being

Cyberbullying research has mostly evolved from psychological researchers (Zych et al., 2015) across the globe who have focused on the impact and correlates of the negative experience on mental health (see Smith, 2019 for a review). One large scale population based study into cyberbullying and adolescent well-being in England (*N* = 110,000 students), found that traditional bullying accounted for greater variability in mental well-being than cyberbullying (Przybylski and Bowes, 2017). However, it concluded that both were associated with poorer mental well-being. Indeed, much evidence indicates that cyber victimization is a predictor of mental health problems, particularly when age and involvement in traditional bullying are accounted for (Kim et al., 2018). For example, such experiences have also been linked longitudinally to depression and anxiety (e.g., Rose and Tynes, 2015; Fahy et al., 2016). In addition, numerous cross-sectional studies have linked cyberbullying involvement to a range of negative psychological outcomes including poorer well-being (Spears et al., 2015), reduced self-esteem (Hinduja and Patchin, 2010), body image dissatisfaction (Ramos Salazar, 2017), Post Traumatic Stress Disorder (PTSD; Ranney et al., 2016) and even psychosis (Magaud et al., 2013).

Such effects of cyberbullying on psychological well-being have in turn been related to a range of negative offline coping behaviors such as increased drug and drink usage (Goebert et al., 2011; Chan and La Greca, 2016) which can in turn place adolescents, especially females at greater risk of assault, sexual assault and forceful sexual relationships (Welsh et al., 2017). From a gendered perspective some studies also indicate that female victims experience higher rates of depression experiencing negative effects from relatively minor or infrequent cyberbullying, and that the effects on their mental well-being can last long after the cyberbullying has ceased (Turner et al., 2013; Selkie et al., 2015).

Nor is it just the victims who are affected. Campbell et al. (2013) investigated a large sample (*N* > 3000) of children and adolescents in Australia and found that cyber bullies had higher scores for conduct problems, hyperactivity, peer problems and emotional problems compared to those not involved in bullying. In addition, a recent systematic review found cyber bullies to be at increased risk of exhibiting suicidal behaviors and worse quality of life compared to non-involved youth (González-Cabrera et al., 2018; John et al., 2018). However, it is the cyber bully/victims that appear to be the most high risk group reporting higher levels of psychological and health issues including post-traumatic stress, mental health impairment, anxiety, self-esteem, academic performance, and depression (Wang et al., 2011; Kowalski and Limber, 2013; Baldry et al., 2018) than either cyber bullies or victims alone.

Although all students involved in cyberbullying are at risk of the effects mentioned, studies show that not all who experience stressors such as bullying exhibit detrimental effects (Hinduja and Patchin, 2007) and can in fact demonstrate positive developmental outcomes in a show of ‘resilience’. As research into cyberbullying continues, an increasing number of global studies have drawn the conclusion that research into factors fostering resilience in every day contexts, may be key to protecting and improving adolescents well-being (Hinduja and Patchin, 2017; Przybylski and Bowes, 2017). Understanding the factors that might increase resilience or protect against the negative effects of cyberbullying is best and most often approached from – a social-ecological system perspective (Papatraianou et al., 2014; Cross et al., 2015). From this perspective, a recent meta-analysis outlined many potential protective factors from the individual to family network which could protect the adverse psychological impact of cyberbullying involvement (Zych et al., 2019).

In a parallel vein, an emerging theme of research has revolved around social factors. This recognizes that more complex issues such as social competence (e.g., Romera et al., 2017), social skills (e.g., Savage and Tokunaga, 2017), social connectedness (McLoughlin et al., 2019) and peer defending (e.g., Lambe et al., 2018) are important for understanding the relationship between cyberbullying involvement and well-being. In particular, one important factor in resilience research is peer friendship and the positive role it can have in buffering against the negative effects of victimization (Kendrick et al., 2012).

### Friendships and Coping

Given the frequency with which studies on cyberbullying mirror those of traditional bullying, it is not surprising that research has demonstrated that young people are more likely to speak to peers about negative online experiences compared to adults, parents, teachers, officials or the authorities (Smith et al., 2008; Jones et al., 2015). Research indicates that peer attachment can be a protective factor against both traditional- and cyber-bullying (Burton et al., 2013), indicating that strong peer attachment may significantly lessen bullying behaviors, with those not involved in bullying reporting considerably higher peer attachment than that of bullies or victims. Further studies indicate, however, that a large diverse group of friends, is not necessarily building resilience, with a lack of association between the number of close friends and levels of depression following traditional bullying (Sapouna and Wolke, 2013). Rather, it is suggested that it is the quality of friendships, and their levels of prosocial behavior, rather than the quantity, that is more important in mitigating such associations as peer victimization (You and Bellmore, 2012) and depression (Kendrick et al., 2012). These studies, however, tend to relate to traditional forms of bullying rather than cyberbullying.

Cyber specific studies appear more varied. For example, a Spanish study of 10-12-year olds indicated that a lack of social skills and difficulties in communicating with peers, which would affect quality of friendship, increased the likelihood of cyberbullying victimization (Navarro et al., 2012). However, other studies show an array of outcomes that appear at variance with these traditional outcomes. For instance, a study of cyberbullying among German adolescents in a classroom context (Festl et al., 2013), showed that real life friendships do not mitigate online victimization. This finding is corroborated by a Hong Kong study of 625 children (Leung and McBride-Chang, 2013) involved in multiplayer video games where online friendships significantly added to pro-social behaviors (e.g., social competence), friendship satisfaction, and self-esteem. However, in a Texan study of high school students, friendship quality did not seem to moderate the negative psychological effects of cyberbullying (Aoyama et al., 2011). These inconsistent findings are attributable to different situational contexts (e.g., age, gender, and friendship quality) in which these studies were carried out. To explain this variation requires further research on whether gender differences in friendship quality are associated with cyberbullying victimization and its psychological consequences.

Therefore, the current study investigated possible associations of gender differences in friendship quality with cyberbullying experiences and psychological well-being. There were three specific aims to this research. The first was to investigate the types of cyberbullying behaviors a large sample of adolescents are engaging in. The second was to determine if there were differences in psychological well-being for cyberbullying involvement, using the Strengths and Difficulties Questionnaire (SDQ) and the Moods and Feelings Questionnaire (MFQ). The third was to explore the association of gender, along with friendship quality and cyberbullying involvement on self-reported well-being of the adolescents. We were not able to determine a formal hypothesis regarding the amount or types of cyberbullying the participants would be engaging in, seeing as there is little available evidence in Ireland on this topic. However, we did hypotheses that there would be some gender differences, in that males would be more likely to engage in cyberbullying through online gaming, as this fact is well-established in the international literature. Furthermore, we hypothesized that higher scores on the well-being measures (the SDQ and MFQ) would be associated with involvement in cyberbullying (as either a victim, bully or both) and lower friendship quality, regardless of gender. Understandably we are limited by the cross-sectional nature of the current study, but we are of the opinion that the benefits in terms of increased understanding outweigh the problems.

## Materials and Methods

### Participants

This study forms part of a wider research project which investigated the prevalence rates of traditional and cyberbullying in Ireland. A brief description of the sample is included here but authors are referred to Foody et al. (2019) for more details on the population and ethical approval. Originally, all post-primary schools in Ireland were invited by email to participate in this study. If interest was noted, the researcher gave more information by email or phone to the principal. Once principals agreed to take part, information and consent forms were sent to the principal to distribute among parents. Principals decided on the classes/age groups to which they would administer the survey, depending on what their own timetable and resources allowed. A final sample of over two thousand participants from 30 different post-primary schools participated (*N* = 2410; 43.2% males and 56.8% females) representing 3.7% of the entire post-primary school population in Ireland. Participants were aged between 12 and 16 years [M(SD): 13.5(1)] and attending 1st to 3rd year in schools across the country.

### Procedure

Once parental consent was obtained, students completed the survey online during school time and in a quiet environment (as determined by school staff). The survey took approximately 30 minutes to complete. Data collection took place between March-May 2017.

### Ethics Statement

This study was carried out in accordance with the recommendations of Dublin City University Research Ethics Committee with written informed consent from all subjects. All subjects gave written informed consent in accordance with the Declaration of Helsinki. The protocol was approved by the above committee. All principals, parents and students were provided with information about the project. Informed and written consent was obtained from principals, students and their parents. Once, principals agreed to complete the survey, they were provided with a link that they could administer to the pupils. All responses were anonymous at the individual and school level and participants were told that they could withdraw from participation at any stage. Thus, answers on the survey could not be traced back to individual students. Both parents and students were advised in their information letter and before completing the survey (for the students) that their answers would be anonymous and completely confidential.

### Survey Instruments

Participants were presented with several questionnaires which are outlined below. In addition, they answered a question on their sex (male/female). Although the question specifically related to sex, it was actually phrased ‘What is your gender?’. We are using the term gender as opposed to ‘sex’ throughout this manuscript. Age and nationality (coded as Irish/non-Irish) was also obtained for all participants. The internal consistency reliability of all the scales and subscales was estimated using both Cronbach’s alpha (α) and McDonald’s omega coefficients (ω), using JASP, a graphical statistical software for common statistical designs (JASP Team, 2019; Love et al., 2019). McDonald’s omega is one of the best alternatives for estimating internal consistency reliability, as it corrects either the underestimation or underestimation bias of Cronbach’s alpha (Revelle and Zinbarg, 2009; Trizano-Hermosilla and Alvarado, 2016).

#### Cyberbullying Questionnaire

In order to assess cyberbullying perpetration and victimization, participants were presented with the Cyberbullying and Online Aggression Scale (Hinduja and Patchin, 2015). Participants were first provided with the following definition of cyberbullying: *“Cyberbullying is when someone repeatedly harasses, mistreats, or makes fun of another person online or while using mobile phones, the Internet or other electronic devices”*. This definition was followed by two initial questions asking participants if they had experienced cyberbullying (victims), or were they perpetrators of such (bullies) in the current school term. Answers included: ‘Never’, ‘Once’, ‘A few times’, ‘Several times’ and ‘Many times’. The scale included two further sections requiring more detail about their experiences with cyberbullying (see Tables 1, 2). The scale required participants to rate the extent and type to which a range of negative experiences had happened to them online (e.g., someone posted mean or hurtful comments about me online) and in which online environments (e.g., in a chat room). Similar answer options were included here: ‘Never’, ‘Once’, ‘A few times’, ‘Several times’ and ‘Many times’. The instrument had good internal consistency for all the subscales. The Cronbach’s and McDonald’s coefficients for the cyber victimization scale were α = 0.90 and ω = 0.90, for the cyber perpetration scale were α = 0.94 and ω = 0.95. For the victimization medium subscale they were α = 0.93 and ω = 0.94, and for the perpetration medium subscale they were α = 0.98 and ω = 0.98. Overall involvement in cyberbullying in *the current term* was categorized into four groups: bully, victim, bully/victim (both a victim and a bully) and non-involved (no involvement in cyberbullying). Response frequencies were coded such that answers from ‘once’ to ‘many times’ was coded as involvement (either as a victim or a bully), while ‘never’ was coded as uninvolved. This is in keeping with previous research using such responses (e.g., O’Moore, 2013).

**Table 1:** Frequencies for each question on the Cyberbullying and Online Aggression Scale.

Questions	Frequency f/% (of sample)	Sig (for gender)	Cramer’s V (for gender)
**Cyber victimization Questions (*N* = 2410)**			
**Someone posted mean or hurtful comments about me online**	325/14.4	<0.001	0.13
Males	86/26.5		
Females	239/73.5		
**Someone posted a mean or hurtful picture online of me**	207/9.2	<0.001	0.1
Males	57/27.5		
Females	150/72.5		
**Someone posted a mean or hurtful video online of me**	72/3.2	<0.01	0.06
Males	19/26.4		
Females	53/73.6		
**Someone created a mean or hurtful web page about me**	35/1.6	<0.01	0.06
Males	6/17.1		
Females	29/82.9		
**Someone spread rumors about me online**	388/17.2	<0.001	0.15
Males	100/25.8		
Females	288/74.2		
**Someone threatened to hurt me through a text/WhatsApp message**	192/8.5	<0.001	0.08
Males	58/30.2		
Females	134/69.8		
**Someone threatened to hurt me online**	214/9.5	<0.01	0.06
Males	72/33.6		
Females	142/66.4		
**Someone pretended to be me online and acted in a way that was mean or hurtful to me**	153/6.8	<0.05	0.04
Males	53/34.6		
Females	100/65.4		
**Cyber Perpetration Questions (*N* = 2410)**			
**I posted mean or hurtful comments about someone online**	83/3.7	>0.05	0.02
Males	30/36.1		
Females	53/63.9		
**I posted a mean or hurtful picture online of someone**	60/2.7	>0.05	0.00
Males	25/41.7		
Females	35/58.3		
**I posted a mean or hurtful video online of someone**	25/1.1	>0.05	0.01
Males	12/48		
Females	13/52		
**I spread rumors about someone online**	75/3.4	>0.05	0.03
Males	25/33.3		
Females	50/66.7		
**I threatened to hurt someone online**	51/2.3	>0.05	0.02
Males	25/49		
Females	26/51		
**I threatened to hurt someone through a text/WhatsApp message**	32/1.4	>0.05	0.01
Males	15/46.9		
Females	17/53.1		
**I created a mean or hurtful web page about someone**	7/.3	>0.05	0.03
Males	5/71.4		
Females	2/28.6		
**I pretended to be someone else online and acted in a way that was mean or hurtful to them**	33/1.5	>0.05	0.04
Males	19/57.6		
Females	14/42.4		


**Table 2:** 4 Medium used for cyber bullying/victimization occurs: prevalence and gender.

Location/place of occurrence	Cyber victimization			Cyber perpetration		
		
	n/%	Significants (for gender difference)	Cramer’s V (for gender)	n/%	Significants (for gender difference)	Cramer’s V (for gender)
**In a chat room**	122/5.4	>0.05	0.02	35/1.6	>0.05	0.02
Males	47/38.5			17/48.6		
Females	75/61.5			18/51.4		
**Through email**	17/0.8	<0.05	0.05	12/0.5	>0.05	0.04
Males	12/70.6			8/66.7		
Females	5/29.4			4/33.3		
**Through instant messages**	177/8.7	<0.001	0.08	33/1.6	>0.05	0.02
Males	49/27.7			16/48.5		
Females	128/72.3			17/51.5		
**Through text message/WhatsApp**	166/7.4	<0.001	0.09	32/1.4	>0.05	0.02
Males	43/25.9			11/34.4		
Females	123/74.1			21/65.6		
**Through mobile phone**	217/9.6	<0.001	0.10	45/2	>0.05	0.03
Males	59/27.2			23/51.1		
Females	158/72.8			22/48.9		
**Through Picture Mail or Video Mail**	42/1.9	>0.05	0.01	14/.6	>0.05	0.01
Males	17/40.5			5/35.7		
Females	25/59.5			9/64.3		
**On Facebook**	138/6.1	<0.01	0.05	34/1.5	>0.05	0.01
Males	44/31.9			16/47.1		
Females	94/68.1			18/52.9		
**On a different social networking website**	186/8.3	<0.001	0.09	32/1.4	>0.05	0.01
Males	50/26.9			15/46.9		
Females	136/73.1			17/53.1		
**On Twitter**	31/1.4	> 0.05	0.01	14/0.6	<0.05	0.05
Males	12/38.7			10/71.4		
Females	19/61.3			4/28.6		
**On Snapchat**	425/20.9	<0.001	0.17	147/7.3	<0.05	0.06
Males	104/24.5			46/31.3		
Females	321/75.5			101/68.7		
**On Yellow**	28/1.2	>0.05	0.00	12/0.5	>0.05	0.04
Males	12/42.9			8/66.7		
Females	16/57.1			4/33.3		
**On YouTube**	46/2	<0.001	0.11	22/1	<0.01	0.06
Males	36/78.3			16/72.7		
Females	10/21.7			6/27.3		
**On Instagram**	245/10.9	<0.001	0.12	63/2.8	>0.05	0.04
Males	62/25.3			20/31.7		
Females	183/74.7			43/68.3		
**In virtual worlds such as Second Life, Gaia, or Habbo Hotel**	26/1.3	>0.05	0.02	11/0.5	<0.05	0.04
Males	13/50			8/72.7		
Females	13/50			3/27.3		
**While playing a massive multiplayer online game such as World of Warcraft, Everquest, GuildWars, Runescape**	80/3.6	<0.001	0.13	38/1.7	<0.001	0.13
Males	61/76.3			34/89.5		
Females	19/23.8			4/10.5		
**While playing online Xbox, playstation, Wii, PSP or similar device**	156/6.9	<0.001	0.20	71/3.2	<0.001	0.18
Males	123/78.8			64/90.1		
Females	33/21.2			7/9.9		


#### Depression

The Moods and Feelings Questionnaire short version (MFQ, Angold et al., 1995; Messer et al., 1995) was used to determine how participants were feeling in the past 2 weeks. Answer options included: not true (0), sometimes (1) and true (2). A higher overall score indicated higher depression. This instrument had good internal consistency in the current study (Cronbach’s and McDonald’s coefficients were α = 0.94 and ω = 0.94).

#### Friendship Quality

A modified version of the Cambridge Friendship Questionnaire was included to investigate the quality of the friendships participants reported having with their peers (Goodyer et al., 1989, 1990). It contained five questions: (1) Are you happy with the number of friends you have? (2) Do your friends know what makes you happy or sad? (3) How often do you see your friends outside of school? (4) Do you talk to your friends about problems? (5) Overall, are you happy with your friends? Response options to the second and fourth question required simple YES/NO answers, while to the first, third and fifth questions they required Likert type answers (i.e., (1) very happy, (2) quite happy, (3) quite unhappy, and (4) unhappy). Regardless of the question type, the response options were considered as continuous such that a higher score was a measure of poorer friendship quality. For example, on question two, an answer ‘No’ indicated poorer friendship quality in the same way as an answer ‘unhappy’ would for question five. Scores were coded and added together such that a *higher* score indicated *poorer* friendship quality. This instrument had internal consistency with Cronbach’s and McDonald’s coefficients of α = 0.60 and ω = 0.65.

#### Psychological Well-Being

The Strengths and Difficulties Scale (SDQ, Goodman, 1997, 2001) is a behavioral screening questionnaire containing statements about psychological attributes relating to five specific subscales. These include behavioral and emotional symptoms (e.g., “I worry a lot”); conduct problems (e.g., “I get very angry and often lose my temper); hyperactivity (e.g., “I am restless, I cannot stay still for long”), peer relationship problems (e.g., “I am usually on my own. I generally play alone or keep to myself”) and prosocial behavior (e.g., I try to be nice to other people. I care about their feelings). Response options are ‘not true’, ‘somewhat true’, and ‘certainly true’. The answer options were coded (0, 1 and 2) and added to give a total score for each individual subscale and an overall difficulties scale that included all subscales except the prosocial behavior. A higher score indicates lower psychological well-being. This instrument had good internal consistency in the current study with Cronbach’s and McDonald’s coefficients of α = 0.76 and ω = 0.77 for the total difficulties scale, α = 0.60 and ω = 0.64 for the conduct problems subscale, α = 0.60 and ω = 0.63 for the hyperactivity subscale, α = 0.70 and ω = 0.77for emotional problems subscale, and α = 0.80 and ω = 0.80 for pro-social subscale. The reliability of the peer problems subscale was, however, not as strong (Cronbach’s and McDonald’s coefficients were α = 0.30 and ω = 0.38.). As such, the peer problems subscale was excluded from further analysis.

### Statistical Analysis

In order to explore our first aim, descriptive statistics were generated for cyberbullying and cyber victimization prevalence as per responses on the Cyberbullying and Online Aggression Scale. Chi square and Cramer’s V were conducted to investigate gender differences on the scale. In order to determine if there were differences in psychological well-being for cyberbullying involvement (aim 2) four categories were created using responses to the global question on cyberbullying relating to involvement in cyberbullying perpetration and victimization in the current school term. These included: bullies, victims, bully/victims (both a victim and a bully) and non-involved (no involvement in bullying). One-way ANOVAs with Bonferroni *post hoc* tests were then generated to compare involvement in cyberbullying and total scores on the MFQ, SDQ and SDQ subscales of emotional problems, conduct problems, hyperactivity and prosocial behavior. The peer problems sub-scale was not included because of its low reliability in the current sample. Finally, when investigating our third research aim, multiple regression analysis using the enter method was conducted to determine the significant factors in determining higher scores on the MFQ, SDQ and the SDQ subscales. All variables were entered into the model, including gender, age, friendship quality, cyber victim, cyber bully and cyber/victim for predicting scores of depression, total difficulties, emotional difficulties, conduct problems, hyperactivity and prosocial behavior.

## Results

### Cyberbullying

Overall involvement in cyberbullying in *the current term* was categorized into four groups: bully (N/% = 34/1.5), victim (N/% = 279/12.4), bully/victim [both a victim and a bully; (N/% = 65/2.9) and non-involved (no involvement in bullying; 1867/83.2%)]. The Cyberbullying and Online Aggression scale contained specific questions relating to how cyber victimization and cyberbullying happened, in addition to the specific medium or apps that it happened on. Participants were asked how often these things happened in the current school year and coding of responses was the same for the general question (above) on cyberbullying involvement. The results are presented in Tables 1, 2.

### Cyberbullying and Psychological Well-Being

A series of one-way ANOVAs found significant differences between the role in cyberbullying involvement and scores on the MFQ [(*F*(3, 2002) = 62.8, *p* = 0.00, partial eta squared = 0.086], total difficulties [(*F*(3, 2005) = 40, *p* = 0.00, partial eta squared = 0.057], emotional problems [(*F*(3, 2030) = 35.96, *p* = 0.00, partial eta squared = 0.05], conduct problems [(*F*(3, 2040) = 21.37, *p* = 0.00, partial eta squared = 0.03]; hyperactivity [(*F*(3, 2040) = 17.2, *p* = 0.00, partial eta squared = 0.025]; and prosocial behavior [(*F*(3, 2037) = 12.99, *p* = 0.00, partial eta squared = 0.019] scales (see Table 3).

**Table 3:** Scores on the well-being measures for four groups of cyberbullying involvement by gender.

Variable	Non-involved *n* = 1686 M(SD)	Cyber bully *n* = 17 M(SD)	Cyber victim *n* = 108 M(SD)	Cyber bully/victims *n* = 19 M(SD)
**Depression**	4.1 (4.5)	4.5 (4.79)	8.09 (5.02)^∗∗∗^	7.87 (5.08)^∗∗∗^
Males	3.15 (4.1)	4.9 (4.7)	7.13 (5.59)	7.67 (5.29)
Females	4.77 (4.68)	4.27 (5)	8.36 (4.74)	6.87 (4.96)
**Total difficulties**	13.22 (5.97)	15.62 (5.99)	17.3 (6.08)^∗∗∗^	17.44 (7.76)^∗∗∗^
Males	13 (6.28)	16.23 (6.3)	15.98 (5.98)	14.53 (6.1)
Females	13.2 (5.65)	15.18 (5.74)	17.76 (6)	17.93 (7.11)
**Emotional problems**	3.67 (2.66)	4 (2.95)	5.52 (2.64)^∗∗∗^	4.79 (3.24)^∗^
Males	2.88 (2.5)	3.4 (3)	4.44 (2.59)	3.1 (2.68)
Females	4.2 (2.59)	5 (2.79)	5.9 (2.3)	5.61 (2.91)
**Conduct problems**	2.37 (1.9)	3.69 (2.2)^∗∗^	3.08 (1.98)^∗∗∗^	3.75 (2.5)^∗∗∗^
Males	2.74 (2.04)	4.06 (2.3)	2.9 (1.87)	3.26 (2.13)
Females	2.05 (1.7)	3.27 (2.1)	3.12 (2)	3.58 (2.4)
**Hyperactivity**	4.44 (2.29)	5.13 (2.19)	5.38 (2.32)^∗∗∗^	5.63 (1.92)^∗∗^
Males	4.6 (2.19)	5.17 (1.8)	4.85 (2.3)	5 (1.7)
Females	4.2 (2.3)	5.17 (1.8)	5.59 (2.3)	5.77 (1.86)
**Prosocial behavior**	7.47 (2.3)	6.07 (2.95)^∗∗^	7.6 (2.19)	5.86 (3.14)^∗∗∗^
Males	6.87 (2.54)	5.41(2.9)	7.3 (2.67)	4.66 (3.39)
Females	7.9 (2)	7.5 (2)	7.8 (1.97)	6.9 (2.44)


*Significant effect when compared to non-involved individuals: ^∗∗∗^*p* = 0.000 ^∗∗^*p* = 0.005 ^∗^*p* = <0.05.*

*Post hoc* tests with Bonferroni revealed significant differences between the four types of bullying involvement and outcomes on the MFQ, SDQ and subscales. Both victims and bully/victims reported significantly higher scores for depression, total difficulties and conduct problems compared to the non-involved (all *p*s = 0.00). Cyber victims also reported significantly more depression (*p* = 0.001) and emotional problems compared to bullies (both *p*s = 0.018) while cyber bullies showed significantly less prosocial behavior compared to victims (*p* = 0.002). Cyber bullies showed significantly more conduct problems compared to non-involved (*p* = 0.002) whereas non-involved students showed significantly more prosocial behavior compared to bullies (*p* = 0.006).

### Friendship Quality

The mean friendship quality score for the overall sample was 7.71 (SD = 2.63). A one-way ANOVA found a significant effect for gender on the friendship quality scale [*F*(1, 2136) = 4.55; *p* = 0.033, eta squared = 0.002] where males (M/SD = 7.86/2.6) reported poorer friendship quality compared to females (M/SD = 7.6/2.61).

A one-way ANOVA was conducted to determine if there were any differences for friendship quality depending on the role in cyberbullying. There was an overall significant main effect [*F*(3, 2161) = 5.158; *p* = 0.001, eta squared = 0.007] with all groups involved in bullying [i.e., victims (M/SD = 8/2.8), bullies (M/SD = 8/2.3) and bully/victims (M/SD = 8.7/3.4)] demonstrating poorer friendship quality compared to the non-involved participants (M/SD = 7.6/2.6). *Post hoc* tests with Bonferroni found a significant difference between friendship quality for the non-involved and bully/victims (*p* = 0.007) but no other significant comparisons (all *p*s > 0.05).

### Associations Between Gender and Friendship Quality on Psychological Well-Being

Multiple regression analyses were conducted to determine the association of friendship quality, gender and involvement in cyberbullying (as either victim, bully or bully/victim) on depression levels, total difficulties, emotional problems, conduct problems, hyperactivity and prosocial behavior (see Table 4).

**Table 4:** Significant predictors of scores on outcome measures using multiple regression.

Variable	*B*	S.E	Beta	*t*	95% confidence interval for B		Significants (*p*)
						
					Lower bond	Upper bond	
**Depression**				
Gender (female)	1.6	0.2	0.16	7.88	1.2	1.9	<0.001
Friendship quality (poorer)	0.39	0.04	0.22	10.34	0.32	0.46	<0.001
Age (older)	0.23	0.1	0.05	2.29	0.03	0.42	<0.05
Cyber victim	3.5	0.3	0.24	11.63	3	4.16	<0.001
Cyber bully/victim	1.03	0.2	0.11	5.13	0.63	1.42	<0.001
**Total Difficulties**				
Age (older)	0.34	0.13	0.06	2.5	-0.08	0.99	<0.05
Friendship quality (poorer)	0.34	0.05	0.15	6.67	0.24	0.44	<0.001
Cyber victim	3.8	0.41	0.2	9.3	3.03	4.6	<0.001
Cyber bully/victim	1.2	0.27	1	4.6	0.7	1.77	<0.001
**Emotional Difficulties**				
Gender (female)	1.45	0.12	0.26	12.53	1.23	1.68	<0.001
Friendship quality (poorer)	16	0.02	0.16	7.5	0.12	0.2	<0.001
Age (older)	0.15	0.06	0.06	2.71	0.42	0.27	<0.01
Cyber victim	1.54	0.18	0.18	8.77	1.19	1.88	<0.001
Cyber bully/victim	0.26	0.11	0.05	2.26	0.034	0.48	<0.05
**Conduct problems**				
Gender (male)	-0.57	0.08	-0.14	-6.53	-0.74	-0.4	<0.001
Cyber bully	0.61	0.18	0.08	3.45	0.26	0.96	<0.01
Cyber victim	0.79	0.13	0.13	6	0.53	1.1	<0.001
Cyber bully/victim	0.46	0.09	0.12	5.3	0.29	0.63	<0.001
**Hyperactivity**				
Gender (male)	-0.22	0.1	-0.05	-2.16	-0.43	-0.02	<0.05
Cyber victim	0.97	0.16	0.14	6.23	0.66	1.27	<0.001
Cyber bully/victim	0.39	0.1	0.09	3.84	0.19	0.59	<0.001
**Prosocial behavior**				
Gender (female)	1.03	0.1	0.22	10	0.83	1.23	<0.001
Friendship quality (stronger)	-0.09	0.02	-0.11	-4.92	-0.13	-0.06	<0.001
Cyber bully (not)	-0.6	0.21	-0.06	-2.89	-1.01	-0.19	<0.01
Cyber bully/victim (not)	-0.5	0.1	-0.11	-4.94	-0.7	-0.3	<0.001


For depression, the resultant model (*R*^2^ = 0.161, adjusted = 0.158; *p* = 0.000) demonstrated that being female, older, having poorer friendship quality, being a cyber victim and a cyber bully/victim were associated with higher depression scores (see Table 4). The significant predictor variables for the other scales are presented in Table 4. All resultant models were significant [total difficulties; (*R*^2^ = 0.083, adjusted = 0.08; *p* = 0.000); emotional problems (*R*^2^ = 0.144, adjusted = 0.141; *p* = 0.000); conduct problems (*R*^2^ = 0.053, adjusted = 0.05; *p* = 0.000); hyperactivity (*R*^2^ = 0.028, adjusted = 0.025; *p* = 0.000); and prosocial behavior (*R*^2^ = 0.035, adjusted = 0.032; *p* = 0.000)]. From Table 4 it can be seen that being female was associated with depression, emotional difficulties and being prosocial while males were prone to conduct problems and hyperactivity problems.

## Discussion

This study was concerned with the association of friendship quality, gender and cyberbullying involvement and the psychological well-being of a large cross-sectional sample of post-primary pupils (aged 12–16 years) in Ireland. The measures used to investigate psychological well-being were the SDQ and MFQ. The results support earlier studies which have examined the impact of cyberbullying on the psychological health of young people involved in bullying either as cyber victims, bullies or bully/victims. For example, the finding that cyber victims as compared to their non-involved counterparts reported more depression, emotional, conduct and hyperactivity problems finds support in much of the existing literature (e.g., Wang et al., 2009; Perren et al., 2010). Similarly, the finding that the cyber bullies as compared to the non-involved demonstrated more conduct problems and were less prosocial supports earlier studies which examined psychosocial risk factors associated with cyberbullying (e.g., Sourander et al., 2010). However, our findings did not fully support those of Campbell et al. (2013) who found, in their Australian sample of 9-19-year olds, that cyber bullies differed from the non-involved on all the SDQ sub scales. Accounting for the differences may be cultural and age differences and the frequency of bullying which the current study did not factor in when examining the SDQ.

As with earlier studies, our cyber bully/victims also demonstrated more depression, total difficulties overall, emotional problems, conduct problems and less pro-social behavior compared to their non-involved counterparts. This is not surprising when one considers the literature which demonstrates these individuals as the highest risk group for a range of internalizing problems (Kowalski and Limber, 2013; Kennedy, 2018). In terms of overall friendship quality, the current results found a significant difference between cyber bully/victims and non-involved students where the latter reported higher friendship quality. Of note, *post hoc* tests did not find significant differences between cyber victims and bullies suggesting that in the current sample of young people, both victims and bullies reported similar friendship quality to youth not involved in bullying.

Friendship quality of the males was poorer than that of the females in the current sample, although it is worth noting that the effect size was very low. The other finding that males across the entire sample had lower prosocial behavior compared to females is perhaps not unexpected in light of earlier research. This points to the impact of gender on children’s lives and in particular on their relationships (Kehily, 2004; Rysst, 2015). Where relationships are specifically concerned, it has been shown that adolescent females who identify with a more traditional feminine gender role are more likely to perceive themselves as using relational aggression than adolescent females who identified with a non-traditional gender role (Crothers et al., 2005). Similarly, other research has shown that males who identify more with traditional masculine gender are more likely to engage in physical forms of aggression as a means of maintaining popularity and status among their peers (Woods, 2009). Recent research suggests that cyberbullying can also provide males with a means to acquire or maintain popularity in early adolescence (Wegge et al., 2016). However, how this manifests itself in relation to the traditional masculine gender needs further evidence.

As seen from Table 1 there were no significant differences between the males and females in the tactics they used to bully their peers. However, Table 2 demonstrates that significant differences were found in the mediums of which cyber victimization and bullying occurred. In respect of victimization, significantly more females than males were found to be subjected to instant messaging, WhatsApp, mobile phones, Facebook, Snapchat, and Instagram, whereas males were more often subjected to bullying on email, YouTube, multiplayer online games and Xbox, PlayStation, Wii, PSP and similar devices. These findings are similar to previous studies where females have been found to be at a higher risk from social networking sites than males (Rey et al., 2018).

Of note, when considering cyberbullying prevalence rates of our sample, our findings in Table 1 provide further support to studies which have indicated that females are at greater risk of cyber victimization than males (Li et al., 2012). Also supporting earlier studies (e.g., O’Moore and Minton, 2009) was our finding that while more males than females admitted to cyberbullying, the differences failed to reach statistical significance. However, of note, was the difference in prevalence rate compared to a recent meta-analysis of cyberbullying for Irish students. The current rate of cyberbullying (i.e., 1.5%) was much lower than that reported by Foody et al. (2017), while the cyber victimization rate was higher (12.4% compared to a pooled estimate of 9.6% for the previous meta-analysis). The higher incidence in victimization may be explained by an increased level of awareness of cyberbullying and the ease with which the ever-increasing variety of mediums can be used to target someone. On the other hand, the lower prevalence rate found in respect of cyber bullies may reflect a greater level of disengagement again due to the increased level of awareness raised through educational programs. It is also worth noting that the same meta-analysis found that there were other factors which influence prevalence rates across studies such as the inclusion or exclusion of a definition of bullying (Foody et al., 2017). It could be argued that the current definition of cyberbullying that was quite general and as such could have led to under-reporting of the phenomenon. However, this is unlikely considering the fact that the participants had to complete the Cyberbullying and Online Aggression Scale which asked details questions about the mediums and modes of cyberbullying and victimization (see Tables 1, 2).

The multiple regression allowed us to determine if scores on the MFQ and SDQ could be predicted by involvement in gender, age, cyberbullying and friendship quality (albeit limited when considering the cross-sectional nature of the study). The results generated particular results for each of the subscales so that we could attempt to scope out which factors might be important to account for scores on the depression, conduct problems, hyperactivity, emotional problems and prosocial behavior scales. In terms of depression, the results suggested that being an older female, having poorer friendship quality, being a cyber victim and cyber bully/victim were all important for higher scores on the MFQ. In parallel, similar variables also predicted higher scores on the emotional subscales. These results are not particularly surprising when one considers the extensive literature base on gender differences in coping styles. Peer socialization along gendered lines begins from infancy with boys, even through use of toys, geared toward problem solving and mechanical tasks, and girls to more pro-social activities, their friendship groups becoming more gender homogenous and reinforcing of social approaches as they grow older (Hanish and Fabes, 2014). In keeping with this, studies have found that women tend to use coping strategies aimed at changing their emotional responses to a stressful situation, while men use more problem-focused methods of handling stressors (Kelly et al., 2008). Poor friendship quality, as mentioned previously does not mean few numbers of friends. Pro-social behaviors exhibited by friends, few or many, who can be bystanders to the cyberbullying, also play a part. Studies show that in regards to cyberbullying only cognitive empathy activation, or mental perspective taking is effective in increasing prosocial bystander behavior in regards to cyberbullying specifically (Barlińska et al., 2015, 2018). That is, not just experiencing another’s emotions, as in affective empathy, but knowing how to put these feelings to use, taking action to not participate, or intervene in the negative online behavior causing those feelings. Given its more active/action focused aspect, it is possible that pro-social behavior, when it comes to females, may correlate with gendered peer socialization. The friends in question do not just need to feel the need to act, but need to be confident in how to act on their empathy. For that reason, anti-bullying programs advocating for increased empathy training may need to incorporate a problem solving element.

Along similar lines, the significant predictors for conduct and hyperactivity problems were being male and involvement in cyberbullying at any level (victim, bully or bully/victim). In this case, age and/or friendship quality were not significant factors in the model. This supports the limited avenue of research which shows that bullying behavior is associated with conduct problems and aggression, particularly among males (e.g., Llola et al., 2016). With that said, it is essential for us to point out that the variables explored (e.g., age, gender, friendship quality and cyberbullying involvement) explained small percentages of the variance in psychological well-being in some cases (e.g., only 2.8% of the variance in hyperactivity was explained by these variables and 3.5% for pro-social behavior). As such, there appears to be many more important variables at play when it comes to determining externalizing behaviors like hyperactivity and conduct problems, particularly, as friendship quality was not a significant predictor variable for determining scores on these measures. It may be that exploring these issues in a separate research stream, as opposed to combined with internalizing behaviors might be one way forward to determine important predictor variables or risk factors. Indeed, the significant variables were better suited to explaining the variance in depression scores (16%) and emotional problems (14.4%). In both cases, poorer friendship quality contributed to this explanation as it has in previous research focusing on internalizing problems within the context of bullying (e.g., Bayer et al., 2018). However, research exploring the role of friendships, bullying/cyberbullying and externalizing issues is less straight forward. Although there is an established link between externalizing problems and bullying involvement (e.g., Boyes et al., 2014; Fite et al., 2014; Hennig et al., 2017), the literature on the role of friendship quality in buffering or mediating this relationship is less well-established. As such we feel this study might make an incremental contribution to the extant literature as we call for more specific and in-depth investigations of cyberbullying along with specific elements of well-being.

That said, there are other limitations to our results which are important to take into account when considering the results presented here. The most obvious limitation is the purely cross-sectional nature of the research which limits the conclusions that can be drawn. Longitudinal research is needed in particular, to parse out the detailed role that friendship quality has in terms of promoting individual resilience and coping skills which may reduce negative mental health outcomes for young people. It is also needed to determine if friendship quality alone does indeed prevent or buffer cyber victimization or if it is only another factor for females and/or individuals of certain age or background. It is also important to point out the low level of Cronbach’s alpha or McDonald’s omega coefficient for the friendship quality questionnaire (α = 0.60 and ω = 0.65). This low coefficient might be due to the YES/NO response option of the second and fourth questions in the questionnaire, given that scale items with two categories may lead to smaller coefficient values compared to those with more than two categories (Peterson, 1994). Nevertheless, a low coefficient value above 0.60 or close to 0.70 can still be considered sufficient reliability for research purposes, while recognizing that it is not ideal for applied settings (Nunnally, 1978; Peterson, 1994). Another limitation is that the results cannot be generalized to the wider population. Although the sample was large, the schools that participated were located across the country and over various socio-economic areas and communities. Going forward, it would be beneficial to draw a population-based sample so that the results could be considered representative of all post-primary pupils in the country.

An avenue for future research might be to investigate the differences between online and offline friendships and their role in buffering the impact of cyberbullying. There are many positives aspects of the internet which include support and friendship groups (with people all across the globe) which some vulnerable individuals might even find more beneficial than interactions offline (Sundberg, 2018). Extant anti-bullying interventions such as KiVa (Kärnä et al., 2011) do focus on promoting friendships and prosocial behavior, along with other elements designed to encourage bystanders to take an active role in bullying reduction. For example, in KiVa students are encouraged to think about ways they can support their classmates to prevent negative experiences like bullying (Salmivalli and Poskiparta, 2012). The use of KiVa is limited in Ireland and no standard anti-cyberbullying program currently exists that all schools draw from. The National Action Plan on bullying (provided by the Department of Education in Ireland) details a set of guidelines and practical steps that principals should follow to prevent and deal with cyberbullying in their schools. However, this is believed to be of limited utility in terms of reducing cyberbullying as it does not direct principals to specific preventative strategies (Foody et al., 2018). Furthermore, it does not provide details around the social and psychosocial factors (e.g., friendship quality) which could be used to enhance current initiatives in the school. Going forward, we argue for principals to consider this research and the wider arena of psycho-social factors when planning and implementing their anti-cyberbullying programs in schools.

## Data Availability

The datasets generated for this study are available on request to the corresponding author.

## Author Contributions

MF designed the study, collected and analyzed the data. MF wrote the first draft of the Materials and Methods, Results, and Discussion. LM wrote the first draft of the Introduction. SK conducted analysis, gave feedback and edited the final draft of the manuscript. JO’HN gave feedback and edited all drafts of the manuscript. All authors edited the final draft.

## Conflict of Interest Statement

The authors declare that the research was conducted in the absence of any commercial or financial relationships that could be construed as a potential conflict of interest.
