# Corrigendum: Friendship Quality and Gender Differences in Association With Cyberbullying Involvement and Psychological Well-Being

**DOI:** 10.3389/fpsyg.2019.02931

**Published:** 2020-01-24

**Authors:** Mairéad Foody, Lian McGuire, Seffetullah Kuldas, James O'Higgins Norman

**Affiliations:** National Anti-Bullying Research and Resource Centre, Dublin City University, Dublin, Ireland

**Keywords:** cyberbullying, friendship quality, gender, psychological well-being, post-primary

In the original article, there was an error. Under the Participants section, a previous publication by the first author was written as BLINDED PUBLICATION. This should read as Foody et al. (2019).

A correction has been made to the **Materials and Methods** section, subsection **Participants**:

“This study forms part of a wider research project which investigated the prevalence rates of traditional and cyberbullying in Ireland. A brief description of the sample is included here but authors are referred to Foody et al. (2019) for more details on the population and ethical approval. Originally, all post-primary schools in Ireland were invited by email to participate in this study. If interest was noted, the researcher gave more information by email or phone to the principal. Once principals agreed to take part, information and consent forms were sent to the principal to distribute among parents. Principals decided on the classes/age groups to which they would administer the survey, depending on what their own timetable and resources allowed. A final sample of over two thousand participants from 30 different post-primary schools participated (*N* = 2410; 43.2% males and 56.8% females) representing 3.7% of the entire post-primary school population in Ireland. Participants were aged between 12 and 16 years [M(SD): 13.5(1)] and attending 1st to 3rd year in schools across the country.”

The reference list has also been updated to reflect this correction.

The authors apologize for this error and state that this does not change the scientific conclusions of the article in any way. The original article has been updated.
